# Intra-host emergence of an enterovirus A71 variant with enhanced PSGL1 usage and neurovirulence

**DOI:** 10.1080/22221751.2019.1644142

**Published:** 2019-07-24

**Authors:** Liang Sun, Aloys Tijsma, Carmen Mirabelli, Jim Baggen, Maryam Wahedi, David Franco, Armando De Palma, Pieter Leyssen, Erik Verbeken, Frank J. M. van Kuppeveld, Johan Neyts, Hendrik Jan Thibaut

**Affiliations:** a KU Leuven, Department of Microbiology and Immunology, Rega Institute for Medical Research, Laboratory of Virology and Chemotherapy, Leuven, Belgium; b Department of Infectious Diseases & Immunology, Utrecht University, Utrecht, the Netherlands; c Department of Imaging & Pathology, KU Leuven, Leuven, Belgium

**Keywords:** Enterovirus A71, SCID, mouse model, CNS, tropism, pathogenesis, receptor

## Abstract

Enterovirus A71 (EV-A71) is one of the main causative agents of hand-foot-and-mouth disease and is occasionally associated with severe neurological complications. EV-A71 pathophysiology is poorly understood due to the lack of small animal models that robustly support viral replication in relevant organs/tissues. Here, we show that adult severe combined immune-deficient (SCID) mice can serve as an EV-A71 infection model to study neurotropic determinants and viral tropism. Mice inoculated intraperitoneally with an EV-A71 clinical isolate had an initial infection of the lung compartment, followed by neuroinvasion and infection of (motor)neurons, resulting in slowly progressing paralysis of the limbs. We identified a substitution (V135I) in the capsid protein VP2 as a key requirement for neurotropism. This substitution was also present in a mouse-adapted variant, obtained by passaging the clinical isolate in the brain of one-day-old mice, and induced exclusive neuropathology and rapid paralysis, confirming its role in neurotropism. Finally, we showed that this residue enhances the capacity of EV-A71 to use mouse PSGL1 for viral entry. Our data reveal that EV-A71 initially disseminates to the lung and identify viral and host determinants that define the neurotropic character of EV-A71, pointing to a hitherto understudied role of PSGL1 in EV-A71 tropism and neuropathology.

## Introduction

Enterovirus A71 (EV-A71) belongs to the family of the *Picornaviridae* and is, together with Coxsackie A viruses, the main causative agent of hand-foot-and-mouth disease (HFMD), which generally affects children below the age of five. EV-A71 is currently comprised of seven genogroups (A-G), each consisting of several subgenotypes. Although EV-A71 usually causes mild and self-limiting disease, several severe and life-threatening complications such as brain stem encephalitis, meningitis, poliomyelitis-like paralysis and pulmonary oedema may also occur [1,2]. EV-A71 mainly causes large HFMD outbreaks in the Asia-Pacific Region [3–5], but the incidence of EV-A71 outbreaks in Europe is increasing [6,7], which is attributed to the rapid emergence of new subgenotypes [8]. Owing to its pandemic potential and the lack of effective antiviral treatments, EV-A71 can be considered a global health threat, especially for young children. To date, two inactivated EV-A71 vaccines have been developed and marketed exclusively in China [9].

EV-A71 is highly contagious and is mainly transmitted via the fecal-oral route. Currently, the gastro-intestinal tract is considered to be the primary site of replication. However, the evidence is accumulating that EV-A71 can also replicate in the respiratory tract and spread via respiratory droplets [8,10,11]. From these primary sites of infection, EV-A71 disseminates and infects other tissues and organs, including the central nervous system (CNS) [12]. Despite years of extensive research, determinants that contribute to the neurotropic character of EV-A71 remain ill defined. Furthermore, increasing evidence suggests a link between EV-A71-induced acute pulmonary oedema and an increase in mortality rates. Although it is currently not yet fully clear whether this pulmonary oedema is a consequence of a virus-induced inflammatory response or rather a neurogenic pulmonary oedema [13,14], evidence is accumulating pointing towards neurogenic involvement with acute, severe destruction of the brainstem tissue as the main contributing factor [15,16].

The viral uncoating receptor SCARB2 and various attachment factors (such as PSGL1, annexin II, DC-SIGN, nucleolin, vimentin, heparin sulphate and sialic acid) have been described to play a role in EV-A71 infection, either by increasing virus attachment or by mediating the uncoating process [17]. However, the exact mechanism by which they contribute to EV-A71-induced disease and outcome severity is still unknown. In addition, although various capsid residues have been described to play a role in determining receptor(s) usage, it remains elusive if and how they contribute to viral dissemination and pathogenesis. For example, a Gln or Gly at position 145 in VP1 has been described to be a determinant of PSGL1 binding [18] and, in epidemiological studies, it has been shown that these residues might also confer a more neurotropic phenotype to the virus [19]. However, no clear evidence on the possible involvement of PSGL1 in neuroinvasion is available so far [8].

Several EV-A71 mouse models have been reported, mostly in suckling mice [reviewed in [20]]. These mice lack a fully matured blood–brain–barrier (BBB) at this stage of development, limiting the relevance of these models for the study of neuroinvasion and the assessment of the potential protective effect of antivirals in the treatment of brain infections [21]. Moreover, in a number of these models, infection is performed by direct intracranial injection, which is cumbersome and not relevant from a clinical perspective [22,23]. In addition, in transgenic mice that express the human SCARB2 receptor, EV-A71 replicates in the CNS but not in the gastro- intestinal or respiratory tracts [24]. Finally, immune-compromised adult mice (i.e. AG129 mice) have been described to be susceptible to EV-A71 infection [25], yet none of the above models have been used to systematically study the distribution and dissemination of the virus within the infected host. We here describe an EV-A71 infection model in severe combined immune-compromised SCID mice that exhibits two different courses of infection: one with a slowly progressing neurological disease following initial infection of the lung compartment, and the other one with fast and exclusive neurological involvement. This dual-course model provides a unique tool to explore tissue and cell tropism of EV-A71, the mechanism behind EV-A71 pathophysiology and in addition, to detail neurotropic determinants.

## Results

### A mouse-adapted EV-A71 strain (812MA) exhibits increased neurovirulence

**Figure 1.**
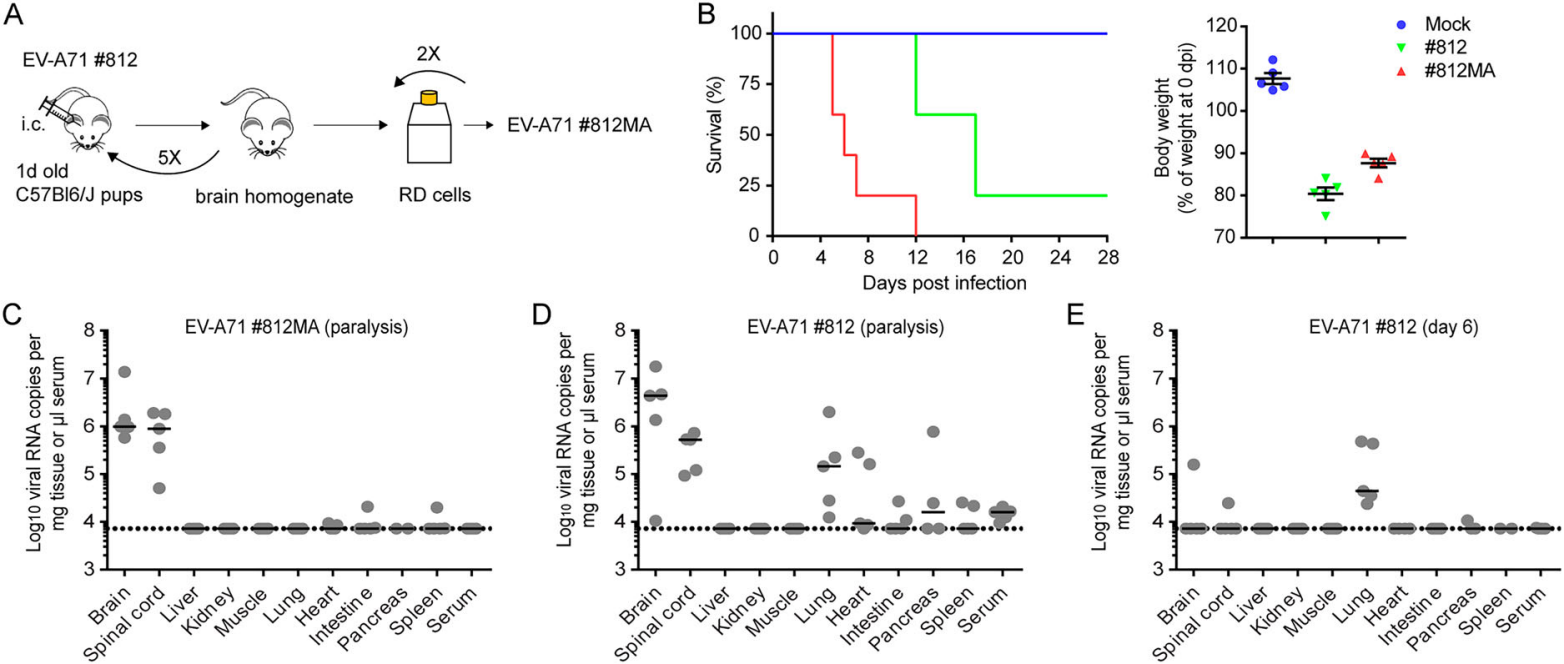
A mouse-adapted EV-A71 strain (#812MA) exhibits increased neurovirulence. (A) *In vivo* adaptation scheme of the clinical isolate EV-A71 #812. EV-A71 #812 was intracranially injected in C57Bl6/J suckling mice (1 d old). Two days post infection (p.i.), mice were sacrificed and the brain homogenate was used to infect new C57Bl6/J 1-day-old-mice. After five passages, the virus was passaged twice in RD cells to obtain the EV-A71 #812MA. (B, left) Survival of mice infected with EV-A71 #812 and EV-A71 #812MA. Six weeks old SCID mice (*n = 5)* were inoculated i.p. with EV-A71 #812, EV-A71 #812MA or PBS (mock) and were monitored daily for signs of disease. (B, right) Relative body weight at the moment of sacrifice. Horizontal bars represent median ± SEM. (C-E) EV-A71 RNA load in various organs. Viral genome copies were determined by means of RT-qPCR (C) in SCID mice infected with 2.4*10^5^ PFU EV-A71 #812MA and sacrificed when paralysis was first observed, (D) 2.4*10^5^ PFU EV-A71#812 and sacrificed when paralysis was first observed, (E) 2.4*10^5^ PFU EV-A71 #812 and sacrificed at six days p.i. (no signs of paralysis). *n = 5* mice for all studies. Horizontal bars represent median ± SEM. The dotted line indicates the detection limit of the assay.

EV-A71 strain #812 (a clinical isolate of subgenogroup C2 obtained from HFMD blisters of an infected child in the UK) was passaged five times in the brains of C57Bl6/J pups and subsequently passaged twice on RD cells to isolate a mouse-adapted (MA) neurotropic variant, EV-A71 strain #812MA (Figure 1(A)). Next, six-week-old severe combined immune-deficient (SCID) mice, which lack functional B cells and T cells, were infected with either the parental #812 strain or the mouse-adapted #812MA strain via the intraperitoneal (i.p) route. Mice were monitored daily and euthanized when paralysis of the hind limbs or >20% weight loss occurred. Mice that were inoculated with 2.4*10^5^ PFU of the parental #812 strain or the #812MA strain showed a median survival of 17 and 6 days, respectively (Figure 1(B)). The outcome of disease correlated with weight loss (Figure 1(B), right). Thus, we obtained an adapted strain, #812MA, with an increased (neuro)virulence compared to its parental #812 strain.

To determine the pattern of tissue tropism of EV-A71 #812MA, SCID mice were inoculated with 2.4*10^5^ PFU and sacrificed when signs of paralysis became apparent (median survival of 6 days) (Figure 1(C)). Subsequently, total RNA was isolated from ten different tissues and viral RNA levels were determined by means of RT-qPCR. In infected/paralysed mice, high RNA levels were detectable in the brain and spinal cord (on average 3.6*10^6^ and 1.0*10^6^ viral RNA copies per mg tissue respectively) as compared to other tissues. In parallel, also α/β and γ IFN receptor-deficient AG129 mice were infected with the same #812MA inoculum (Suppl. Fig. 1). In contrast to SCID mice, infected/paralysed AG129 mice showed a non-restricted systemic infection with high viral loads in almost all organs, which is less reminiscent of the natural tropism of EV-A71 in humans suggesting that SCID mice may represent a more valuable model than AG129 mice to study tissue tropism of EV-A71. Next, we determined the tissue tropism of the parental #812 strain when signs of paralysis became apparent (median survival of 17 days) (Figure 1(D)). Interestingly, infection with the parental strain revealed a more dispersed tissue tropism than #812MA, showing not only high viral RNA levels in the brain and spinal cord, but also in other organs such as lung and heart tissue (5/5 mice). To compare the pathogenesis without the bias of a longer viral persistence in the system, we also determined the tissue distribution of #812 at day 6, the day of euthanasia for most of the #812MA-infected mice (no neurological symptoms were observed at that time) (Figure 1(E)). Interestingly, high viral RNA levels were only observed in the lungs (on average 2.1*10^5^ per mg tissue), whereas viral RNA could only be detected in the brain and spinal cord of one mouse. Together, these data indicate that the tissue tropism in SCID mice differs dramatically between EV-A71 strains #812MA and #812. The lung compartment is most likely the primary site of replication of #812 following intra-peritoneal inoculation, whereas #812MA exhibits an exclusive neurotropism.

### Amino acid I135 in the VP2 “puff” loop is critical for neurotropism in mice

**Figure 2.**
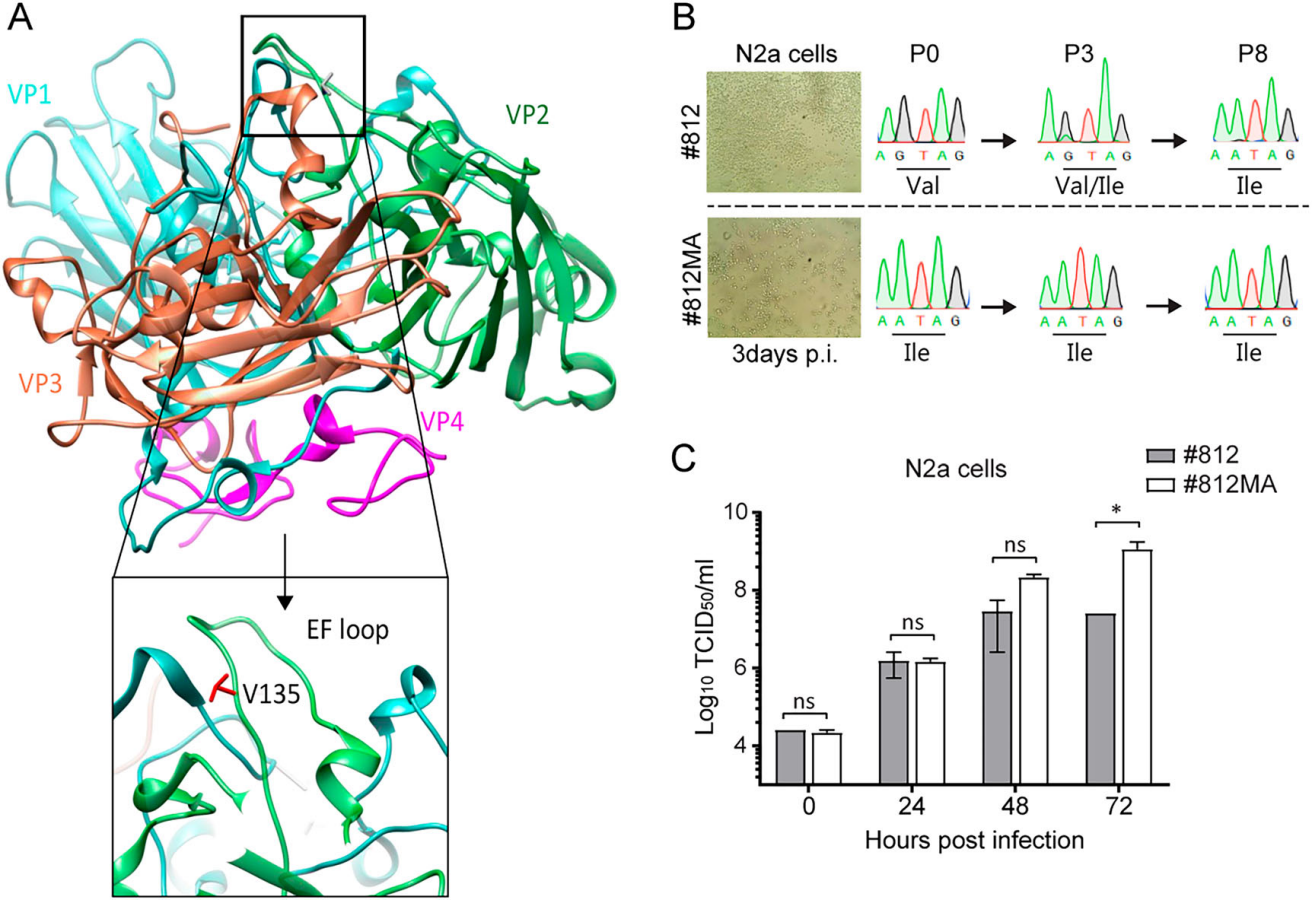
VP2 substitution V135I in mouse-adapted EV-A71 (#812MA) is a genetic determinant of neurotropism (A) Side view of a protomeric structure of EV-A71 (3VBS) along the 2-fold symmetry axis (USCF Chimera). The four capsid proteins VP1-4 are depicted in blue, green, red and purple, respectively. A close up view of the EF loop and amino acid V135 of VP2 (in red) is also shown. (B) Scheme of EV-A71 #812 *in vitro* adaptation. EV-A71 #812 was used to infect neuroblastoma murine N2a cell line (MOI = 1). Three days p.i., supernatant (diluted 1:5 was used to infect a fresh monolayer of N2a cells). At passage 0, 3 and 8, viral RNA was extracted and VP2 was sequenced. (C) Replication kinetics of EV-A71 #812 and #812MA on N2a cells. At the indicated times p.i., N2a cells were harvested and viral levels were determined by means of end-point titration on RD cells. Error bars represent the mean ± SEM of 3 biological replicates. *P* values were calculated by two-way ANOVA in GraphPad, *P < .05; ns, *P* > .05.

To find genetic determinants of the neurotropic phenotype, we fully sequenced EV-A71 #812MA and #812 genomes isolated from the respective virus stock (inoculum) and selected organs (lung, brain and spinal cord). Only one mutation was identified in the inoculum of #812MA, at position 135 in the surface-exposed highly variable EF-loop (residues 128–148, also called the “puff”) of structural protein VP2 (Table 1, Figure 2(A)). In particular, #812 carried a valine (Val^135^) at this position, whereas #812MA carried an isoleucine (Ile^135^). Virus isolated from the brains and spinal cords of all #812MA-infected mice carried VP2 Ile^135^ (5/5 mice). Strikingly, VP2 Ile^135^ was already present in virus isolated from the brains and spinal cords of one #812-infected mouse prior to the onset of neurological symptoms (day 6) and in five #812-infected mice (5/5 mice) at the day of paralysis. In contrast, the virus isolated from the lung compartment of #812-infected mice all carried VP2 Val^135^ at day 6 (5/5 mice) and was present in almost all mice at the day of paralysis (4/5 mice) (Table 1). These results strongly suggest that, by acquiring VP2 Ile^135^, the virus adopts the ability to spread to and replicate in neuronal tissues. To address whether the acquisition of VP2 Ile^135^ results from a host adaptation and/or tissue adaptation, we passaged the #812 strain (VP2 Val^135^) in various human (including RD and two neuroblastoma cell lines) and mouse cell lines (including L929 fibroblasts and N2a neuroblastoma cells). Interestingly, following replication in N2a cells, #812 acquired VP2 Ile^135^ as a minority variant by passage 3 and as an established substitution by passage 8 (Figure 2(B)). In contrast, passaging of #812 in all other human and mouse non-neuronal cell lines did not result in amino acid changes (Suppl. Fig. 2), suggesting that VP2 Ile^135^ results from both host and a tissue adaptation. In addition, #812MA exhibited a moderate replication advantage in N2a cells as compared to #812 (Figure 2(C)). Altogether, these data suggest that one amino acid change at position 135 of VP2 is necessary and sufficient for the virus to replicate in the central nervous system (CNS) of SCID mice.

**Table 1.** VP2 amino acid substitutions in EV-A71 #812 and #812MA.

Virus	Paralysis		Day 6
	EV-A71 #812MA	EV-A71 #812	EV-A71 #812
Inoculum	I135	V135	V135
Brain	I135 (5/5)	I135 (5/5)	I135 (1/5)
Spinal cord	I135 (5/5)	I135 (5/5)	I135 (1/5)
Lung	I135 (5/5)	I135 (1/5)	V135 (5/5)
		V135 (3/5)	
		V135 + M149 (1/5)	

Amino acid substitutions in VP2, compared to the inoculum of EV-A71 #812 and #812MA are indicated. The ratio of samples positive for the particular amino acid over the total number of samples is indicated between brackets.

### (Motor)neurons and type II pneumocytes are two possible cell targets for EV-A71

**Figure 3.**
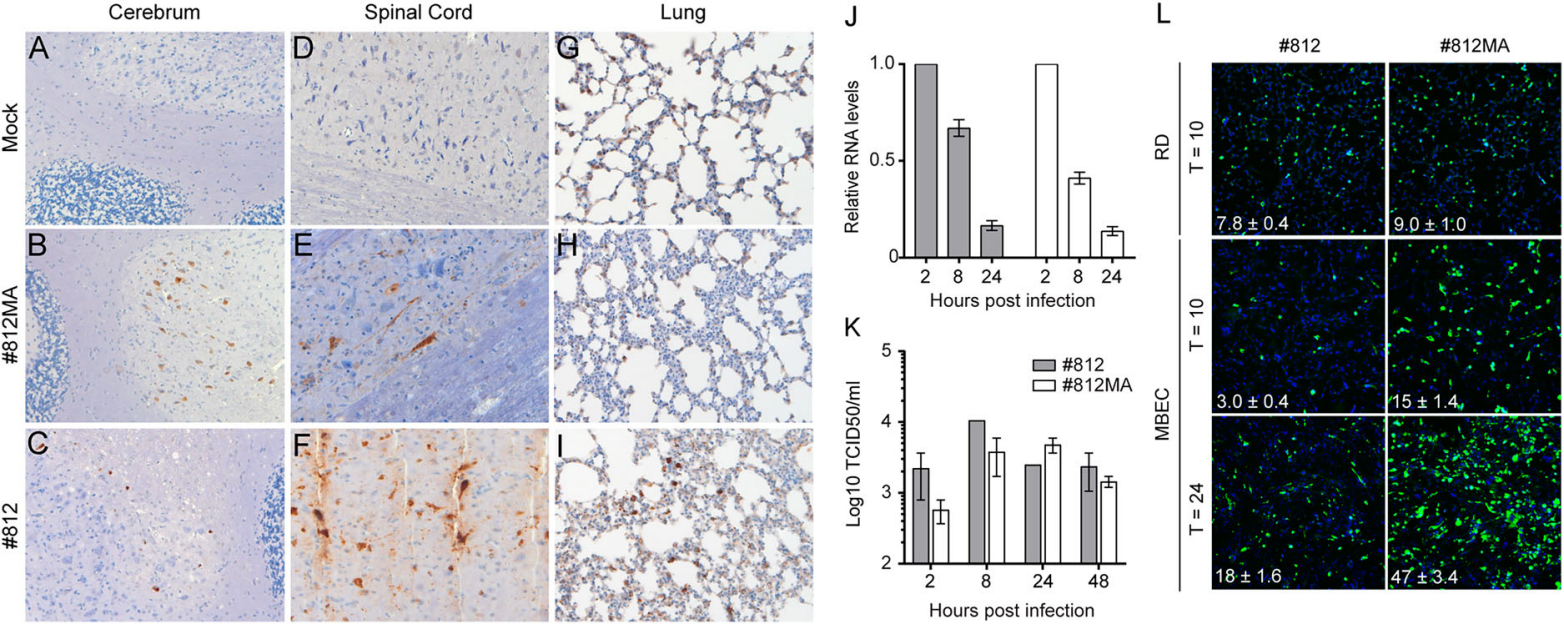
EV-A71 infects (motor)neurons and type II pneumocytes. EV-A71 antigen expression in SCID mouse tissue. Mice were either mock infected or infected with EV-A71 #812MA or EV-A71 #812 and sacrificed when paralysis was first observed. Paraffin embedded, formalin fixed tissue sections were stained with an anti-EV-A71 antibody. Representative pictures of (A-C) cerebrum, (D-F) spinal cord and (G-I) lungs. (J-K) Infection of bone-marrow-derived macrophages. Cells were differentiated in M-CSF-containing medium for 6 days and infected with #812 and #812MA (MOI = 1). At selected times p.i., cells were harvested. Infection was quantified by RT-qPCR (J) and TCID_50_ determination on RD cells (K). (L) MBEC and RD cells were infected with EV-A71 #812 or EV-A71 #812MA for 1 h. Following fresh medium replacement, the cells were further incubated for the indicated times. After fixation, the infected or mock cells were stained by dsRNA antibody, followed by high-content imaging counting. Images shown are representative fluorescent micrographs. The data shown are mean ± SEM of one (J-K) or two (L) independent experiments each containing three (J-K) to six (L) biological replicates.

The development of a model with a complex pathophysiology during infection prompted us to investigate the specific cell tropism of both EV-A71 #812MA and the parental EV-A71 #812 in different infected tissues by means of immunohistochemistry (IHC). In the CNS of infected mice with signs of paralysis (median day 6 p.i. for #812MA and day 17 p.i for #812), viral antigens were detected in neurons (Figure 3(A–F)). More specifically, in the brain, mainly neurons of the cerebellum stained positive (Figure 3(A–C)) whereas in the spinal cord, viral antigens were exclusively detected in motor neurons (Figure 3(D–F)). This is in line with the pathophysiology reported in EV-A71 infected individuals with neurological complications. Other cells of the CNS such as glia cells did not stain for viral antigens. No viral antigens were detected in liver, kidney, muscle, heart, or spleen, whereas viral antigens were observed in the exocrine compartment of the pancreas and in the intestines of one mouse (Suppl. Fig. 3A). In parallel, H&E staining on brain and lung tissue of #812 and #812MA-infected mice revealed no clear evidence of virus-induced lesions or infiltration of immune cells (Suppl. Fig. 3B).

Intriguingly, IHC analysis on the lungs of #812-infected SCID mice revealed the presence of EV-A71 antigens in type II pneumocytes as well as in alveolar macrophages (Figure 3(G–I)) again pointing to a possible involvement of the lung compartment in supporting EV-A71 #812 replication. To investigate whether macrophages support EV-A71 #812 replication, various murine and human macrophages cell lines (Suppl. Fig. 4A) and murine bone-marrow-derived macrophages (Figure 3(J–K)) were infected with #812 and #812MA. None of these cell types could support the replication of either EV-A71 #812 or EV-A71 #812MA as assessed by RT-qPCR (Figure 3(J)) and end-point titration (Figure 3(K) and Suppl. Fig. 4A). Thus, our data suggest that macrophages do not support initial EV-A71 replication. In addition, to exclude the potential role of macrophage in neuroinvasion, mice were treated with clodronate liposomes via the intranasal and the intraperitoneal route to deplete macrophages locally and systemically. The local depletion was verified by IHC on lung tissue at 48 h post clodronate treatment (Suppl. Fig. 4B). SCID mice were concomitantly infected with EV-A71 #812 and weight and signs of paralysis were monitored. No clear differences were observed between the macrophage-depleted or control mice (Suppl Fig. 4C), suggesting that macrophages do not play a role in virus spread to the CNS. Together, our data point towards a role of type II pneumocytes as the primary cell target of EV-A71 #812 (Val^135^ variant) in the murine respiratory tract.

We next assessed whether a virus-mediated destruction of the BBB may provide a possible explanation for the observed neuroinvasive character of #812MA. To this end, we compared the infection efficiency of EV-A71 #812 and #812MA in MBEC and CMEC/D3 cells, mouse and human brain endothelial cells, respectively. Interestingly, #812MA showed an increased replication efficiency (Figure 3(L) and Suppl. Fig. 5A) and induction of CPE (Suppl. Fig. 5B) as compared to #812 in MBEC cells, but not in CMEC/D3 or other human neuronal cell lines. In parallel, we also tested the susceptibility of human endothelial cells derived from the umbilical cord. No replication advantage of #812MA as compared to #812 could be observed (Suppl. Fig. 5A). Together, our data demonstrate that the neuropathology induced by #812MA (Ile^135^ variant) might be a consequence of replication in (motor) neurons and that the virus might invade the CNS by direct infection and more extensive destruction of the BBB.

### Substitution V135I enhances PSGL1 usage by mouse-adapted EV-A71

**Figure 4.**
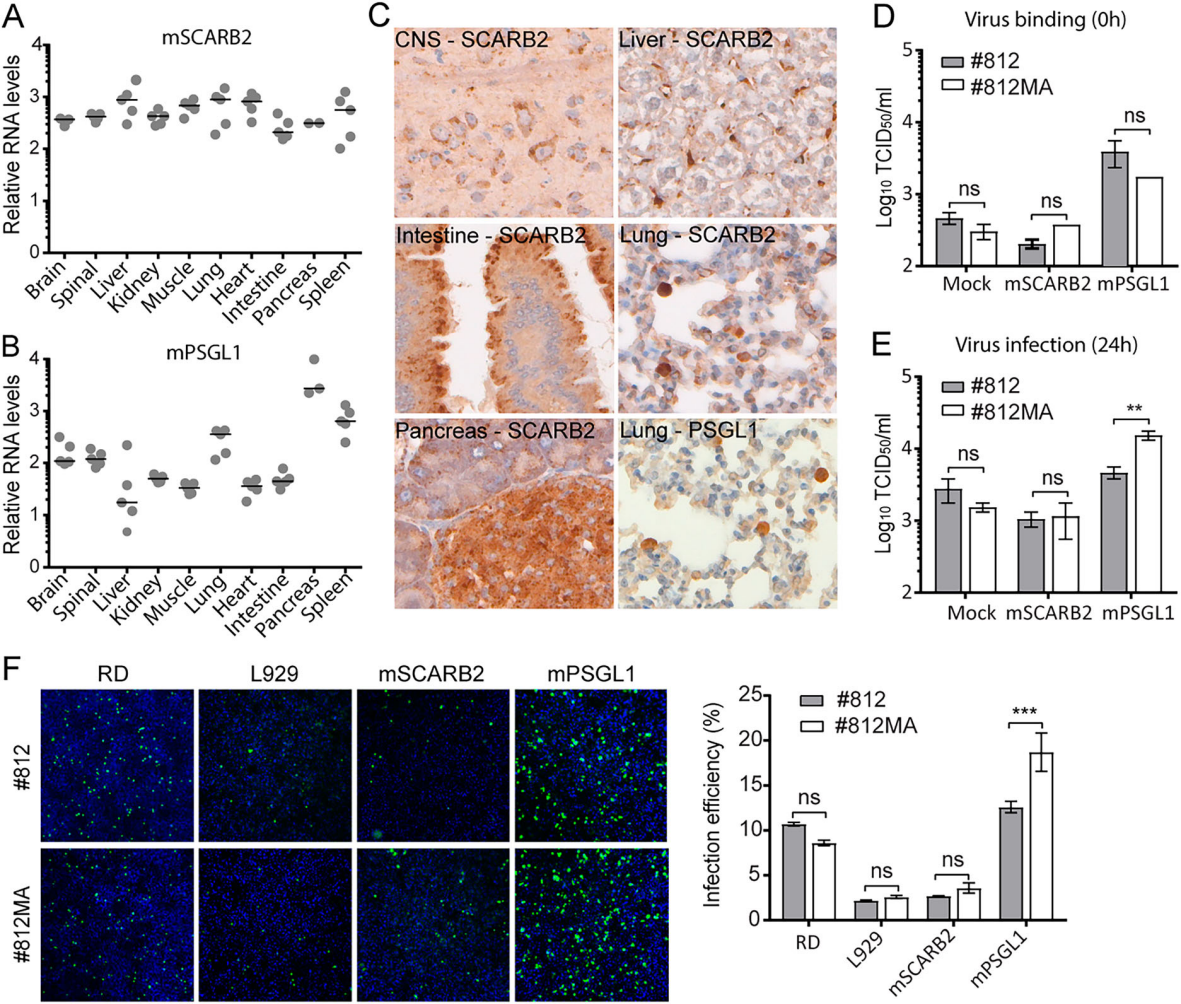
Substitution V135A enhances PSGL1 usage by mouse-adapted EV-A71. The relative levels of mRNA of (A) SCARB2 and (B) PSGL1 in ten different tissues was quantified by means of RT-qPCR. Data were normalized to β-actin (housekeeping gene). RT-qPCR data are expressed as relative levels. Horizontal bars represent the mean. (C) SCARB2 and PSGL1 in various organs tissues assessed by means of immunohistochemistry. SCARB2 protein expression in CNS, intestine, pancreas, liver and lung. (D-E) Infection of EV-A71 #812 and #812MA in L929 cells transfected with mouse SCARB2 and PSGL1 expression plasmids; Cells were infected with #812 and #812MA 24 h post transfection and frozen at 0 (D) or 24 (E) hours p.i. Following three freeze-thaw cycles, infectious virus titres (CCID_50_/ml) were determined by means of end-point titration on RD cells. (F) Mouse SCARB2 and PSGL1-transfected L929 cells or RD cells were infected with #812 and #812MA; L929 cells were fixed at 48 h p.i., RD cells at 24 h p.i., followed by dsRNA (green) and nuclei (blue) staining. Images shown are representative fluorescent micrographs. The data shown are mean ± SEM of two (F) or three (D-E) independent experiments each containing three (D-E) or four (F) biological replicates. *P* values were calculated by two-way ANOVA in GraphPad, **P < .01; ***P < .001; ns, *P* > .05.

As receptors are often important determinants of tropism and pathogenesis, we next assessed the levels of the two main EV-A71 receptors, SCARB2 and PSGL1, in different tissues. Gene expression levels were determined by means of RT-qPCR (Figure 4(A–B)) and protein expression was visualized by means of IHC (Figure 4(C)). The SCARB2 gene was found to be evenly expressed in all tissues (Figure 4(A)) whereas for PSGL1 a more variable gene expression profile was observed with higher levels in the lung, CNS, pancreas and spleen (Figure 4(B)). At a cellular level, the SCARB2 protein was mainly expressed on motor neurons of the CNS, the epithelial cells of the intestine, the islets and exocrine cells of the pancreas and liver Kupffer cells (Figure 4(C)). In lungs, both SCARB2 and PSGL1 antigen were found in type II pneumocytes and/or macrophages. These data further corroborate the hypothesis that motor neurons in the CNS and type II pneumocytes in the lung are the target cells for EV-A71 infection.

To investigate a possible involvement of mouse SCARB2 and PSGL1 during entry of EV-A71 #812 and #812MA, mouse PSGL1 and SCARB2 expression plasmids were transiently overexpressed in poorly permissive L929 cells, followed by EV-A71 #812 or #812MA infection. Overexpression of mPSGL1, but not mSCARB2, greatly increased virus particle attachment of both #812 and #812MA to L929 cells (Figure 4(D)). In addition, despite comparable virus input (as determined on RD cells, in which Ile^135^ does not enhance infection), #812MA proved to be more infectious than #812 in L929 cells overexpressing mPSGL1, as shown by an increased infectious virus production (Figure 4(E)) and dsRNA staining (Figure 4(F)). On the contrary, no replication advantage was noted in L929 cells overexpressing mSCARB2 (Figure 4(E–F)), despite successful transfection (Suppl Fig. 6A). Interestingly, both viruses showed comparable replication kinetics in L929 cells expressing hSCARB2 and hPSGL1 (Suppl Fig. 6B), showing that the adaptation of #7812MA to mPSGL1 did not affect the usage of hPSGL1. Finally, to explore whether mouse PSGL1 also plays a role in infection of MBEC cells we performed neutralization experiments using an anti-mouse PSGL1 antibody. The antibody was however not able to neutralize #812, #812MA and the negative control mengovirus (Suppl Fig. 6A). Altogether, these results reveal that the amino acid change Ile^135^ in VP2 leads to an enhanced usage of mPSGL1 and support the hypothesis that mPSGL1 might play a role in defining tissue tropism in our mouse model.

## Discussion

Progress in understanding EV-A71 induced pathogenesis has been hampered by the lack of reliable small animal infection models with a relevant pathophysiology. We here established an EV-A71 infection model in SCID mice to study the dynamic distribution and tissue tropism of an EV-A71 clinical isolate. Using this model, we identified that EV-A71 initially disseminates to the lungs and showed that by acquiring a single residue mutation in the viral capsid the virus adopts the capacity to spread to and infect the CNS thereby causing neurological disease. This restricted tissue tropism of EV-A71 in SCID mice is in sheer contrast with the non-restricted infection that we and others observed in AG129 mice. These results corroborate previous findings on the importance of type I and III IFN innate defence to restrict EV-A71 infection [25,26]. Similarly, it has been shown previously for poliovirus that unequal IFN responses define tropism and pathogenicity [27]. In addition, the complex pathophysiology as well as the straightforward readout makes this a very useful platform to assess the *in vivo* efficacy of novel antiviral strategies.

EV-A71 antigens were detected in (motor) neurons of the spinal cord and the cerebellum in all paralysed mice. This observation is a specific trait of neuronal EV-A71 infections in humans and may explain why EV-A71 can cause paralysis and balancing problems. It is currently unclear how EV-A71 bypasses the BBB to infect the CNS. A number of studies suggest that EV-A71 either hijacks leukocytes to pass the BBB or infects the CNS via retrograde axonal transport [28,29]. Our data suggest a model in which EV-A71 enters the mouse brain by directly destroying endothelial cells and inducing leakage of the BBB. Furthermore, we showed that EV-A71 initially infects the lungs, and more specifically type II pneumocytes, after which the virus disseminates to the CNS. This slowly progressing neuropathology following initial infection of the lung supports the current idea that, in humans, EV-A71 initially replicates in the lungs and from there disseminates systemically and infects other organs [8,10,11]. Our small animal model could thus constitute an invaluable tool for future studies to gain more insights into virus dynamics and to fully characterize determinants that allow infection of the lung and CNS.

A key determinant of virus tropism and the associated pathogenesis is the distribution of its cognate receptors in different tissues. Human SCARB2 was shown to be a functional EV-A71 uncoating receptor and transgenic mice expressing hSCARB2 develop similar pathological features as humans [17]. In contrast, it is still unclear how PSGL1 supports EV-A71 infection as transgenic mice expressing hPSGL1 were not susceptible to EV-A71 infection [24,30]. We identified a single residue (Ile^135^) in VP2 that enhances the usage of mPSGL1 and that increases the neurotropic character of EV-A71. This mutant was previously described to develop as a consequence of host adaptation in murine fibroblast cells, but this only after a minimum of 60 passages [31]. However, in our hands, the Ile^135^ VP2 variant was in a murine neuroblastoma cell line already selected after 8 passages. This was not the case in various human cell lines or a non-neuronal murine line. This suggests that this variant is preferably selected in neuronal tissue and further underscores the possible involvement of Ile^135^ VP2 in the neurotropism of EV-A71 in the murine host. Reminiscent of the tropism of poliovirus, viral antigens are exclusively detected in the motor neurons of the spinal cord. Although PSGL1 is primarily expressed in leukocytes, expression in neuronal tissue has also been reported [32]. It remains to be studied whether in our model the presence of PSGL1 drives infection of motor neurons and whether differences in expression patterns between motor neurons and other types of neuronal cells determine susceptibility to infection. Moreover, it is currently still under investigation whether PSGL1 drives EV-A71 infection of endothelial cells of the BBB. Furthermore and in line with previous results, our data indicate that EV-A71 cannot engage mSCARB2 for infection [33,34], putting our model forward as an unique tool to study the hitherto understudied role of PSGL1 in mediating EV-A71 tropism and neuropathogenesis.

Residue Ile^135^ in VP2 is part of the so-called EF loop or “puff loop” [35], which is exposed at the surface of the viral capsid and resides at the southern rim of the viral “canyon”. Another residue of the VP2 puff loop, residue 149, was previously shown to be important for replication in L929 cells overexpressing human PSGL1, pointing to a possible involvement of the puff loop in the interaction with mouse PSGL1 during viral entry [36]. It is generally believed that clusters of positively charged amino acids (e.g. Lys^244^) at the 5-fold vertex of the EV-A71 capsid are critical for attachment to sulphated tyrosine residues on the amino terminus of human PSGL1 [37]. The fact that we did not observe any differences in the levels of #812 and #812MA virus particles bound to L929 cells overexpressing mPSGL1 suggests that both strains might have similar abilities to bind mPSGL1. In line, we did not identify any differences in the 5-fold vertex between both strains. Importantly, #812 was able to use mPSGL1 to infect cells, albeit to a lesser extent than #812MA. Together, this suggests that the improved infection by #812MA might be a consequence of an improved internalization or uncoating efficiency rather than an increased binding capacity. It has been shown recently in an EV71–SCARB2 complex structure that EV-A71 engages its cellular receptor hSCARB2 via the VP2 EF loop [38]. It remains to be established whether EV-A71 can engage mPSGL1 or a hitherto unknown murine uncoating factor via the same region and initiate uncoating via similar structural rearrangements as was observed for human SCARB2. Additionally, both EV-A71 #812 and #812MA carry residue Glu^145^ in VP1, which has been associated with an impaired binding of hPSGL1. We demonstrated that, despite the presence of this residue, both strains can replicate efficiently in L929-hPSGL1 cells (in contrast to the non-PSGL1 binder BrCr strain) (Suppl Fig. 6B), suggesting that VP1 residue 145 may not be the only determinant for hPSGL1 usage. The fact that antibodies against mouse PSGL1 cannot neutralize #812 and #812MA infection of susceptible MBEC cells can likely be explained by the fact that these antibodies do not bind to the epitopes that interact with the virus. An alternative explanation may be that the infection of MBEC cells occurs via alternative mechanisms that do not involve the usage of mouse PSGL1.

Altogether, this study provides not only novel insights into the mechanism underlying neurotropism, neurovirulence and dissemination of EV-A71 but also offers an invaluable tool to study the *in vivo* efficacy of antivirals thereby accelerating the development of antiviral therapies against this important pathogen.

## Materials and methods

### Cells, viruses and reagents

Human rhabdomyosarcoma (RD) cells, human neuroblastoma (SK-N-SH, SH-SY5Y) cells, murine neuroblastoma (N2a), murine cells fibroblast (L929), murine macrophage (RAW264), murine brain endothelial (MBEC) cells, murine Lewis lung carcinoma (LCC) cells were maintained in DMEM (Gibco) supplemented with 10% heat-inactivated foetal bovine serum (FBS), 1% sodium bicarbonate (Gibco) and 1% L-glutamine (Gibco). L929-hSCARB2 cells and L929-hPSGL1 cells are kindly provided by Prof. Satoshi Koike (Tokyo Metropolitan Institute of Medical Science, Japan) and were grown in DMEM supplemented with 10% FBS, 1% sodium bicarbonate, 1% L-glutamine and 10 µg/ml puromycin. Human endothelial cells (hCMEC/D3 and HUVEC) are kindly provided by Prof. S. Liekens and Dr. M. Benkheil and were maintained in EBM-2 medium supplemented with 1 ng/ml human basic fibroblast growth factor, 10 mm HEPES, 1% chemically defined lipid concentrate, 5 μg/ml ascorbic acid, 1.4 μM hydrocortisone, 1% pen–strep and 5% of heat-inactivated FBS. EV-A71 strain H08300 461 (#812) of sub-genogroup C2 was obtained from European Virus Archive and was isolated from HFMD blisters of an infected-child in UK. EV-A71 #812MA (Mouse Adapted variant) was obtained during this study. EV-A71 BrCr strain, was obtained from the National Institute of Public Health and the Environment (RIVM), Bilthoven, The Netherlands. EMCV strain Mengo was a kind gift from Prof. T. Michiels (de Duve Institute, Université catholique de Louvain, Belgium). For *in vitro* assays the medium used was supplemented with 2% FBS. Mouse SCARB2 and PSGL1 cDNA clone expression plasmids containing a signal peptide and anti-mouse PSGL1 antibody (50770-RP01) were purchased from Bio-connect.

### Mouse adaptation of EV-A71 #812

All experiments were performed according to the university guidelines for animal care and are approved by the ethical committee of the University of Leuven. One-day-old C57Bl6/J mice were intracranially (i.c.) infected with 20 µl of EV-A71 #812. Two days p.i., mice were euthanized, brain tissue was collected and homogenized and it was used to infect naïve one-day-old C57Bl6/J i.c. The experiment was repeated five times (five passages) and 100 µl of the P5 homogenate was used to infect RD cells. After two passages in RD cells, a viral stock was harvested and used for the following experiment EV-A71 #812MA.

### Mouse infection studies and tissue collection

SCID mice (in-house bred) were housed in individual ventilated cages. Mice were intraperitoneally (i.p.) injected with EV-A71 #812 or EV-A71 #812MA at an indicated concentration (PFU/mouse). Limb paralysis and/or >20% weight loss were used as a criterion for early euthanasia by Nembutal injection. After euthanasia and extensive perfusion with PBS, indicated organs/tissues were collected. Each organ/tissue sample was cut in half; one half was frozen in liquid nitrogen for subsequent viral RNA isolation and the other half was fixed with 4% formaldehyde for immunohistochemistry.

### Viral RNA isolation and RT-qPCR

Frozen tissues were homogenized in lysis buffer using a Precellys 24 homogenizer. After centrifugation of the homogenates, the supernatants were used for RNA isolation (Qiagen RNeasy). Viral RNA load in the tissues was quantified by means of RT-qPCR using the One-step RT-qPCR MasterMix Low ROX (Eurogentec) in a Lightcycler 96 (Roche). A serial dilution of synthetic DNA (gBlocks, IDT) corresponding to the sequence/size of the amplicon was used to build a standard curve. For the expression of PSGL1 and SCARB2 relative gene expression was calculated using the 2^-ΔΔCt^ method [20]. β-actin was used as internal control.

### Serial passaging

EV-A71 #812 and EV-A71 #812MA were used to infect N2a, L929, RD, SK-N-SH and SH-SY5Y cells (MOI = 1). Following 1 h infection at 37°C and a PBS washing step, cells were further incubated for three days. The infected cell cultures were freeze/thawed twice. For all subsequent rounds of infection in the virus was 1/5 diluted.

### Histology

Formalin fixed, paraffin embedded tissue sections were deparaffinized using Bond Dewax Solution (Leica) after which Epitopes were retrieved using Bond Epitope Retrieval Solution 1 (Leica). The sections were stained for EV-A71 antigen (10F0, abcam), PSGL1 (bs-0561R, Bioss) or SCARB2 (NB400-129, Novus). In addition, haematoxylin and eosin staining was performed to visualize inflammation.

### Macrophage isolation

Bone marrow from the femur of two SCID mice was collected. Cells were filtrated and kept on ice. A suspension of 2 × 10^6^ cells/ml was prepared in differentiation medium: RPMI, 10% FBS, 1X non-essential amino acids, sodium pyruvate, PenStrep and 20 ng/ml of M-CSF. On day 1, 3, and 6, the medium was refreshed with pre-warmed new differentiation medium. On day 7, macrophages were fully differentiated and ready for infection (MOI = 1). At indicated times p.i. the supernatant from infected-macrophages was used to determine viral titres by end-point titration on RD cells. Viral RNA was obtained after extraction of total RNA from infected-macrophages at indicated times p.i. and quantified by RT-qPCR.

### Macrophage depletion *in vivo*

To allow the specific depletion of macrophages in the lung compartment, SCID mice were treated with 200 µl of clodronate liposomes or PBS liposomes: 100 µl were injected intraperitoneally (i.p.) and 100 µl were administered intranasally after anaesthesia (xylazine\ketamine\atropine). Concomitantly, mice were infected with 10^6^ PFU of EV-A71 #812 i.p. Macrophage depletion from the lung compartment was confirmed by immunohistochemistry (two days post clodronate treatment). Infected mice were monitored daily for the sign of disease and euthanasia was administered whenever 20% of weight loss or paralysis of the limbs was observed.

### Infectivity and immunofluorescence assays

Cells were infected with virus for 1 h at 37°C, followed by a PBS washing step. Cells were supplied with fresh medium, and incubated for the indicated time-points. For infectivity assays, cells were subjected to freeze/thaw cycles and viral titres were determined by end-point dilution on RD cells. For immunofluorescence assays, cells were fixed by 3.7% paraformaldehyde for 15 min at room temperature. Fixed cells were stained by 1:500 diluted anti-dsRNA monoclonal antibody (J2, SCICONS). The number of infected cells were examined and quantified by high-content imaging.

## Supplementary Material

Supplemental Material
