# Supplementary figures and images for: Intra-host emergence of an enterovirus A71 variant with enhanced PSGL1 usage and neurovirulence
Source: Emerg Microbes Infect. 2019 Jul 24;8(1):1076–85. doi: 10.1080/22221751.2019.1644142 (PMC6711088; doi:10.1080/22221751.2019.1644142)

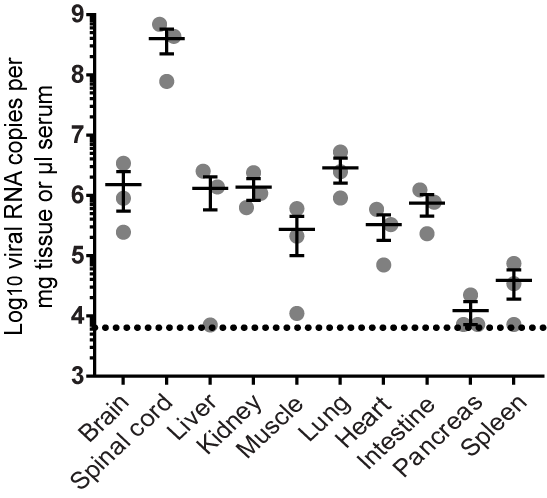

Supplement: Supplemental Material [file TEMI_A_1644142_SM0227.zip › Suppl Fig 1.tif]

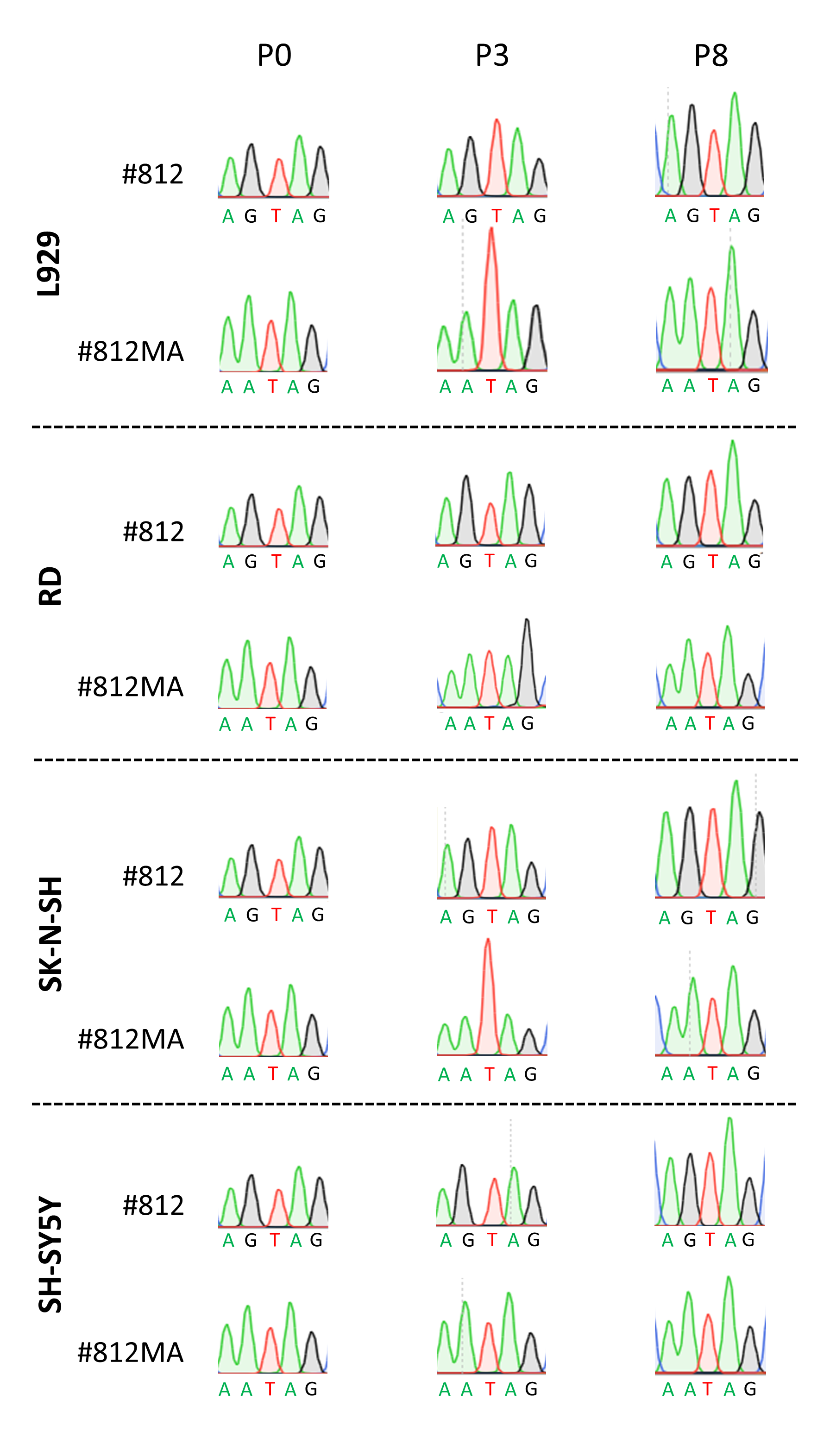

Supplement: Supplemental Material [file TEMI_A_1644142_SM0227.zip › Suppl Fig2.tif]

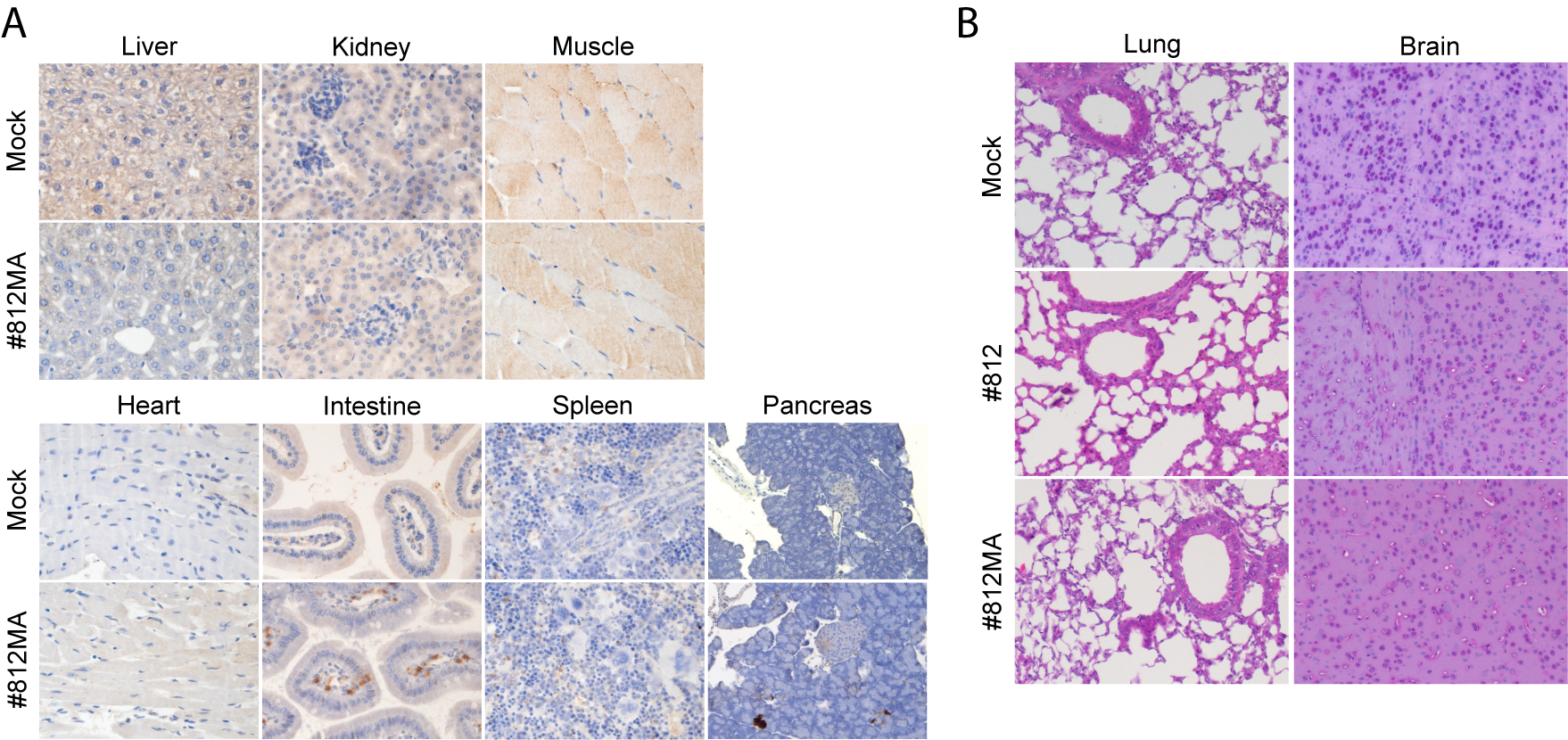

Supplement: Supplemental Material [file TEMI_A_1644142_SM0227.zip › Suppl Fig3 - revised.tif]

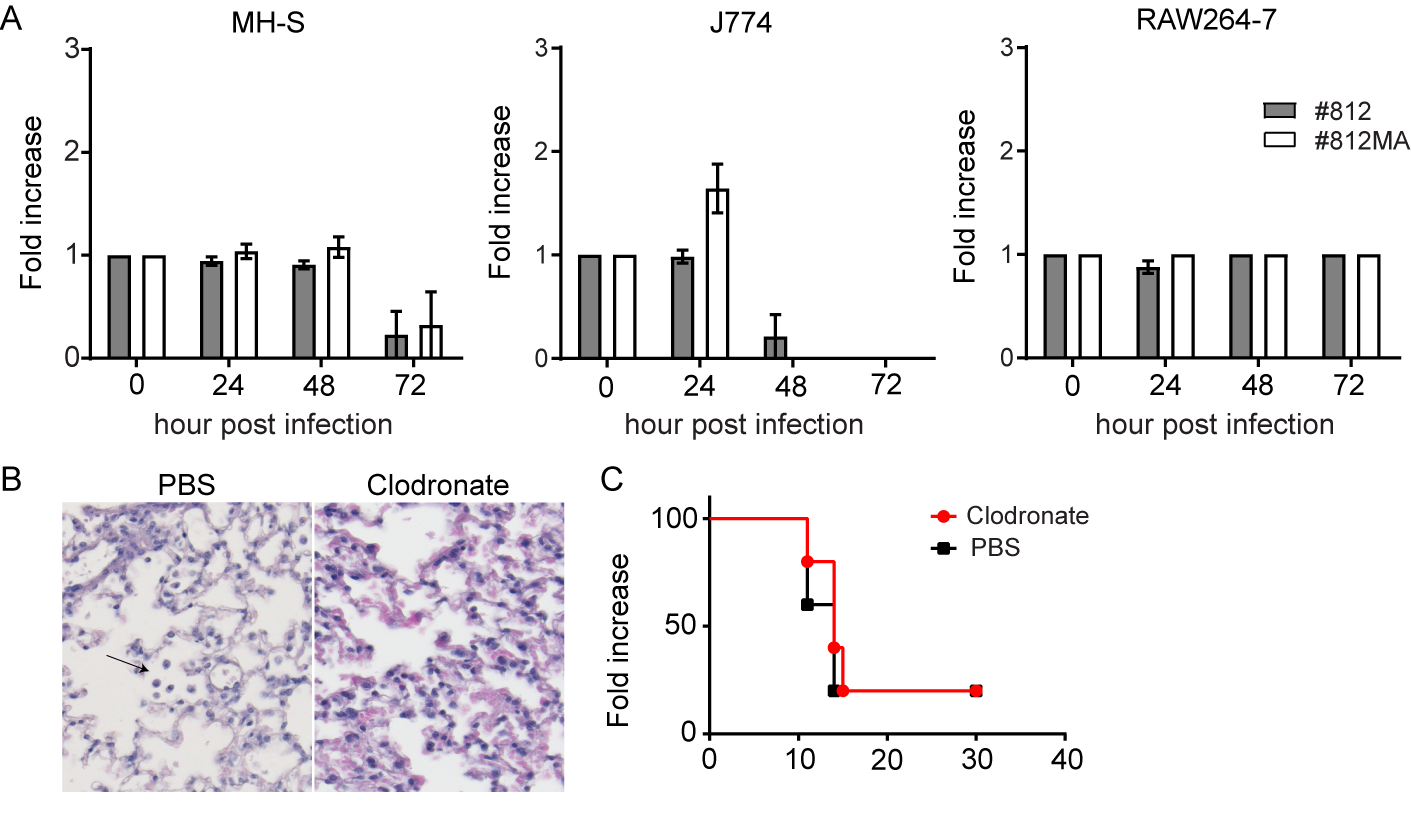

Supplement: Supplemental Material [file TEMI_A_1644142_SM0227.zip › Suppl Fig4.tif]

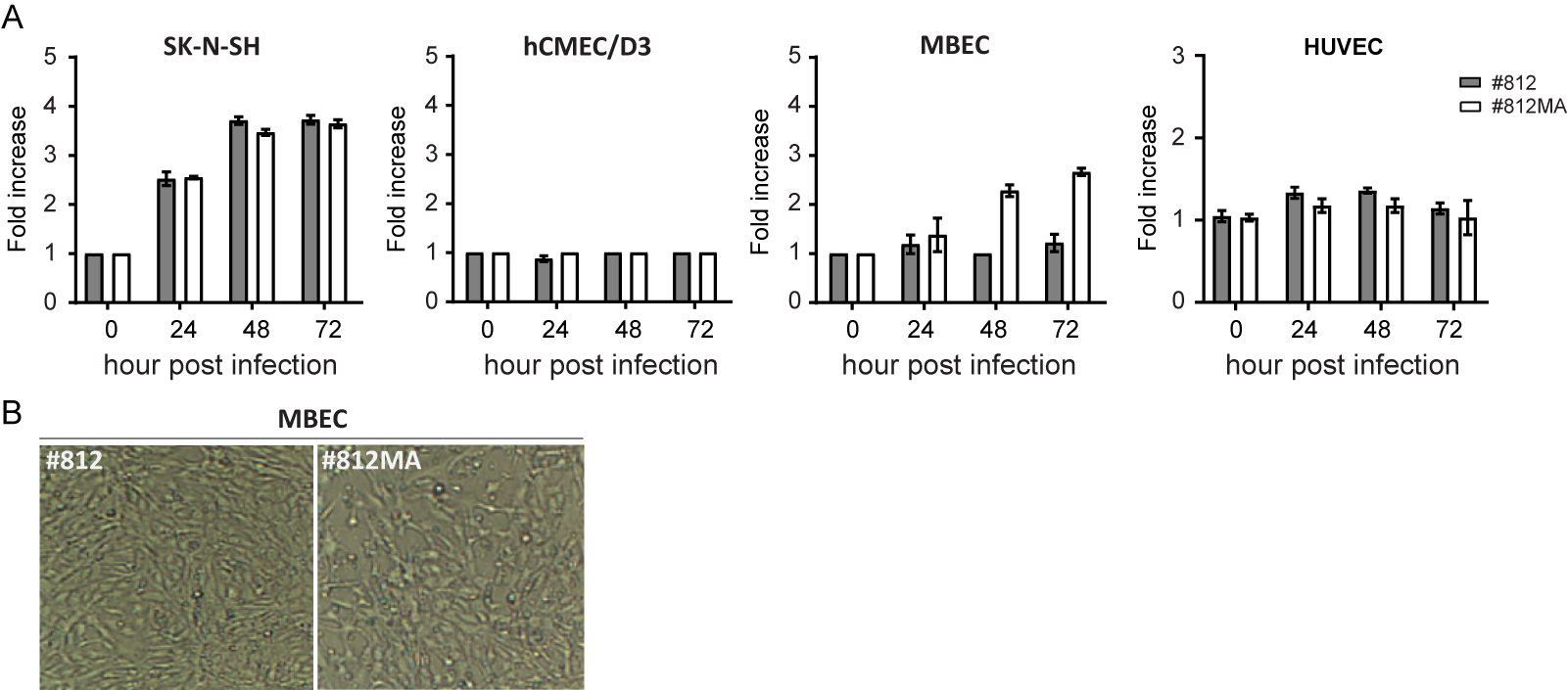

Supplement: Supplemental Material [file TEMI_A_1644142_SM0227.zip › Suppl Fig5 - revised.tif]

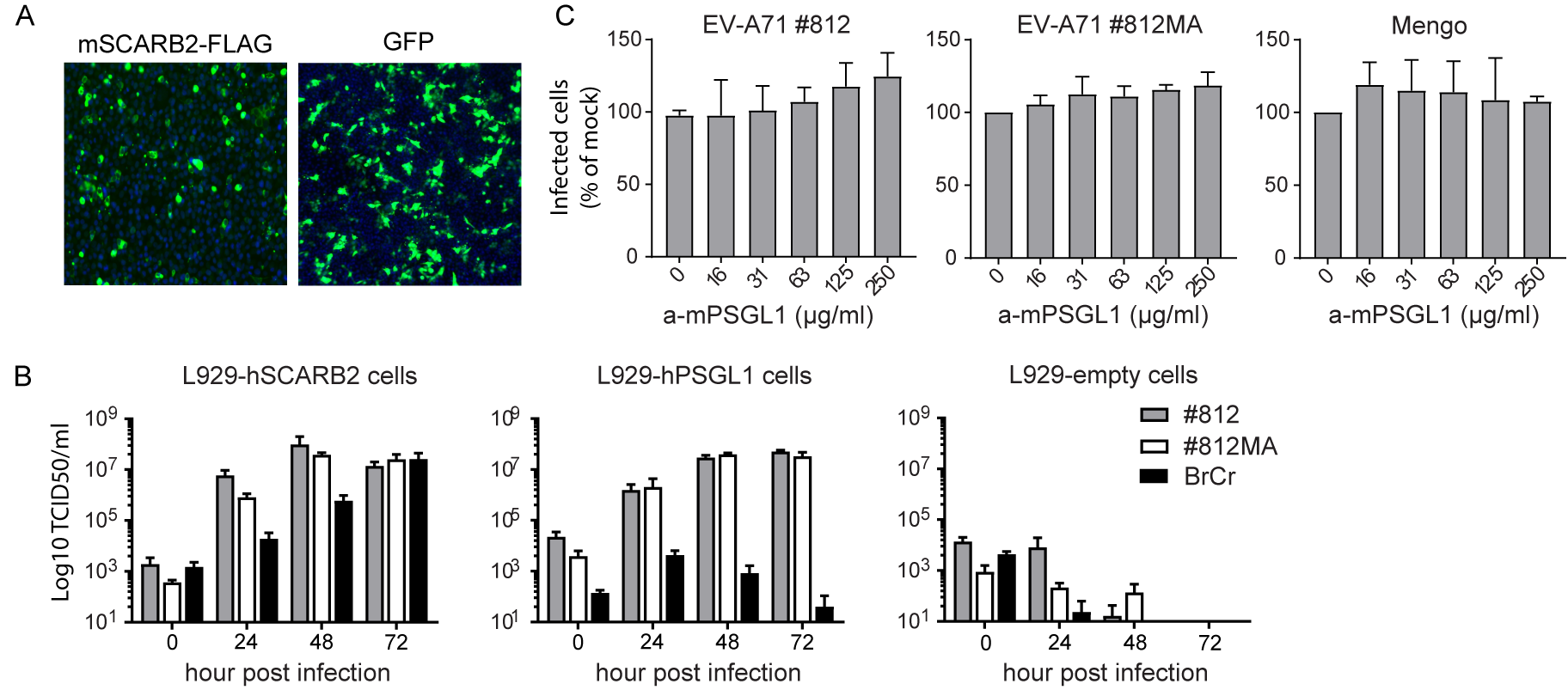

Supplement: Supplemental Material [file TEMI_A_1644142_SM0227.zip › Suppl Fig6 - revised.tif]
